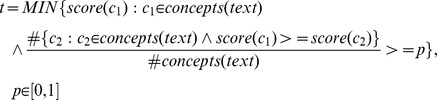# Enhancement of Chemical Entity Identification in Text Using Semantic Similarity Validation

**DOI:** 10.1371/journal.pone.0062984

**Published:** 2013-05-02

**Authors:** Tiago Grego, Francisco M. Couto

**Affiliations:** Departamento de Informtica, Faculdade de Cincias, Universidade de Lisboa, Lisboa, Portugal; Rensselaer Polytechnic Institute, United States of America

## Abstract

With the amount of chemical data being produced and reported in the literature growing at a fast pace, it is increasingly important to efficiently retrieve this information. To tackle this issue text mining tools have been applied, but despite their good performance they still provide many errors that we believe can be filtered by using semantic similarity. Thus, this paper proposes a novel method that receives the results of chemical entity identification systems, such as Whatizit, and exploits the semantic relationships in ChEBI to measure the similarity between the entities found in the text. The method assigns a single validation score to each entity based on its similarities with the other entities also identified in the text. Then, by using a given threshold, the method selects a set of validated entities and a set of outlier entities. We evaluated our method using the results of two state-of-the-art chemical entity identification tools, three semantic similarity measures and two text window sizes. The method was able to increase precision without filtering a significant number of correctly identified entities. This means that the method can effectively discriminate the correctly identified chemical entities, while discarding a significant number of identification errors. For example, selecting a validation set with 75% of all identified entities, we were able to increase the precision by 28% for one of the chemical entity identification tools (Whatizit), maintaining in that subset 97% the correctly identified entities. Our method can be directly used as an add-on by any state-of-the-art entity identification tool that provides mappings to a database, in order to improve their results. The proposed method is included in a freely accessible web tool at www.lasige.di.fc.ul.pt/webtools/ice/.

## Introduction

Areas such as genomics and proteomics have embraced large-scale experimental surveys and free and openly accessible reference databases, which contain structured information about biomedical entities such as genes and proteins. In chemistry this is not always the case, since large-scale experimentation has been conducted primarily by the pharmaceutical industry, and thus a vast amount of data is proprietary and not openly accessible. Because of this, scientific literature is still a common way to report chemical data. However, chemical data recently started to be publicly available with the release of database resources such as PubChem [1], ChEBI [2] and even combined ones [3], [4]. These databases mostly represent a structured version of a part of the knowledge present in chemical literature, such as scientific research papers and patent documents. Thus, the process of automatically retrieving and extracting chemical knowledge is of great importance to aid the development and growth of chemical databases.

This process of gathering data from the literature for compiling information in databases usually requires expert curators to manually analyze and annotate the literature [5], and is being used in diverse fields including protein interaction networks [6], neuroanatomy [7] and has been the standard in the chemical domain [8] although this is a tedious, time consuming and costly process [9]. Fortunately, text mining systems have already shown to be helpful in speeding up some of the steps of this process, namely performing named entity recognition and linking the recognized entities to a reference database [10]–[12]. Text mining for entities such as genes and proteins has been extensively evaluated with promising results [13], and some tools such as Textpresso [14] and Geneways [15] have been successfully used in support of database curation tasks. Chemical text mining is gathering increasing interest by the community, but despite the potential gains still faces significant challenges [16], [17]. Most common methodologies applied to the problem of chemical named entity recognition include dictionary and machine learning based methods.

Dictionary based approaches require domain terminologies to find matching entities in the text and depend on the availability and completeness of these terminologies. An advantage of this approach is that entity resolution is directly obtained by the name entity recognition task, since each entity recognized is inherently linked to an individual term of the terminology. However recognition is limited to the data that exists in the used terminology and given the vast amount of possible chemical compounds, the terminologies are always incomplete. A popular text processing system that uses a dictionary based approach for identifying a wide variety of biomedical terms, including chemicals, is Whatizit [18]. This system finds the entities by dictionary-lookup using pipelines, each based on a specific terminology. One of the available pipelines is based on ChEBI and allows for the recognition and resolution of ChEBI terms.

Machine learning based approaches require an annotated corpus which is used to build a model that can be applied in the named entity recognition of new text. Systems using this approach use named entity recognition as a classification task that tries to predict if a set of words represent an entity or not. The bottleneck of this approach is the availability of an annotated corpus large enough to enable the creation of an accurate classification model, and the need for an entity resolution module for mapping the recognized entities to database entries. An example of a machine-learning based chemical entity recognition system uses CRF models to locate the chemical terms [19] and a lexical similarity method to perform resolution of those terms to ChEBI [20].

The existing fully automated tools are still far from providing perfect results to fulfill the requirements and expectations of databases curators [21], [22]. This paper is a step forward in improving the results provided by any text mining system trying to identify chemical entities in literature. This improvement is achieved by our novel validation method that takes the outcome of a text mining system and checks its coherence in terms of ontological annotation [23]. The underlying assumption behind our method is that a text (e.g. paragraph, abstract, document) will have a specific scope and context, i.e. the entities mentioned in that text have a semantic relationship between them. This assumption is based on the fact that authors only mention two chemical entities in the same fragment of text if they share a semantic relationship between them. The implementation of our validation method is then based on measuring the chemical semantic similarity of the identified chemical compounds as a means to discriminate validated entities from outliers, i.e. entities unrelated to the other entities also identified nearby.

Semantic similarity has been extensively applied using several biomedical ontologies, notably the Gene Ontology (GO), for which several semantic measures have been developed and discussed [24]. While GO contains terms for describing proteins, ChEBI contains terms that describe chemical compounds. Proteins can be described as a set of GO terms the same way a compound can be described as a set of ChEBI terms. One concept frequently used in semantic similarity measures is the information content (IC), which provides a measure of how specific and informative a term is. The IC of a term *c* is quantified as the negative log likelihood:
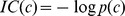
where p(c) is the probability of occurrence of *c* in a specific corpus, estimated by its frequency.

Resnik’s similarity measure [25] is a commonly used node-based measure where the similarity between two term is given simply by the IC of their most informative common ancestor (MICA):
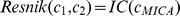



The measure simUI is an example of a edge-based measure [14]. Given two compounds 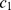 and 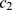, the set of all ancestral terms up to the root node from and including 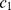 and 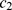 are 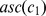 and 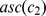 respectively, simUI is defined as the number of terms in the intersection of 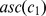 with 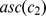 divided by the number of terms in their union:
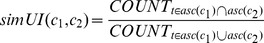



The measure simGIC [26] is a hybrid measure that uses IC in addition to the graph structure and is defined as the sum of the IC of each term in the intersection of 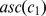 with 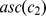 divided by the sum of the IC of each term in their union:
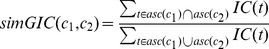



Chemical semantic similarity was previously adopted with success in a work that aimed to improve compound classification [27].

We applied our validation method to the annotations provided by two text mining tools, representing two distinct approaches, when applied to a gold standard patent document corpus. The entities found by those tools in the text were used as input to our method. The idea was to verify if our method was able to improve the precision by filtering the outlier entities and by validating the entities with strong semantic relationships. The results show the feasibility of our method, since it significantly increased precision with a small impact on recall. For example, it was able to increase precision in more than 25% by only discarding 6% of the correctly identified entities.

We will start by detailing and discussing the results obtained by the proposed method and in the following section we describe the tools, data and methods applied.

## Results and Discussion

### Patent Corpus

Manually annotated documents are essential for the development and evaluation of text mining systems. Thankfully, a corpus of forty patent documents was manually annotated with ChEBI concepts by a team of curators from ChEBI and the European Patent Office in an effort to promote the development of chemical text mining tools (http://chebi.cvs.sourceforge.net/viewvc/chebi/chapati/patentsGoldStandard/). This gold standard was afterwards enriched with mappings of the manually annotated chemical entities due to the fast growing of the ChEBI database [7] and the enriched version of the corpus can be found in the website of the web tool that includes our method (www.lasige.di.fc.ul.pt/webtools/ice/).

### Text Mining Results

Two distinct methods for entity recognition and resolution were applied to this patent corpus. One of them is a dictionary method, Whatizit [18], that performs ChEBI term lookup in input text. The other is a machine-learning method that uses an implementation of CRF (Conditional Random Fields) [20.

The output of chemical text mining systems consists of chemical entities recognized and mapped to ChEBI (automatic annotations). These automatic chemical annotations are the input for our validation method. Table 1 presents an outline of the entity recognition and resolution results obtained for both text mining systems in the patent corpus. We can see that for the same corpus the dictionary-lookup method recognized and mapped to ChEBI almost 18,700 putative chemical entities, while the CRF-based method only recognized and mapped to ChEBI about 10,700 putative chemical entities. However, the amount of identified entities that turned out to be true positives is similar for both methods (about 4,600 entities) when considering an exact matching assessment. This means that the CRF-based method has a higher precision, having for instance for exact matching a 44.8% precision while the dictionary-lookup method only obtains 24.3%.

**Table 1:** Automatic entity identification results.

Method	Annotations	TP	Precision	Recall
Dictionary	18,683	4,530	24.3	46.7
CRF-based	10,681	4,783	44.8	49.3

Results of entity identification (recognition and resolution to ChEBI) obtained by the two used tools in the patent corpus. An exact matching assessment was considered. Annotations indicate the total amount of entities recognized, TP indicates how many were in accordance to the gold standard.

### Validation Results

The list of ChEBI concepts identified by a text mining system in a given fragment of text is the input of our validation method. For each input ChEBI concept, our method measures the semantic similarity between it and all the other ChEBI concepts in that list. We used different semantic similarity measures, namely Resnik, SimGIC and SimUI. Our method then returns for each concept the list of most similar concepts sorted by their similarity value. We defined the validation score of a given concept as the similarity value of the most similar concept returned by our method. The validation score measures our confidence that the concept has been correctly identified by the text mining system. Next, our method ranks the input list of ChEBI concepts using their validation score, and a threshold can be defined in order to split the ChEBI concepts in consistent entities (when its validation score is higher than the defined threshold) and outlier entities (when the validation score is below the defined threshold).

**Figure 1:**
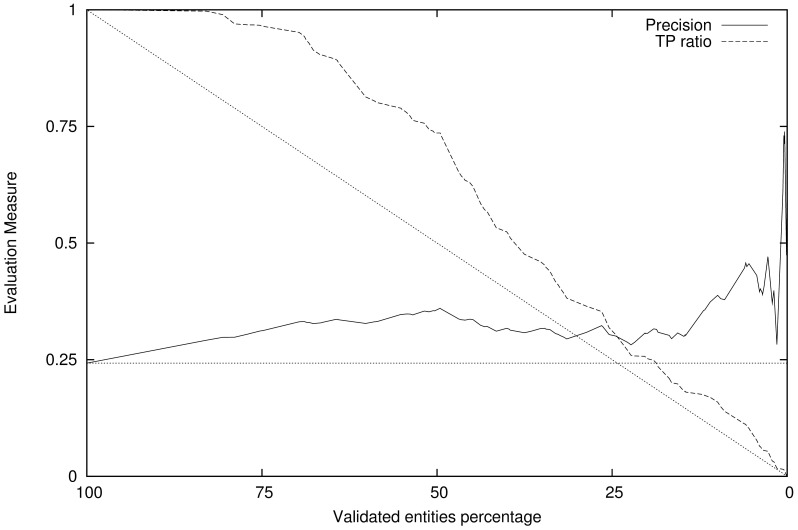
Validation of Whatizit annotation results. Shows the variation in precision and recall with the validation score threshold, using the Resnik measure with a document as text window. Straight dots represent the expected behavior of a random validation system.

**Figure 2:**
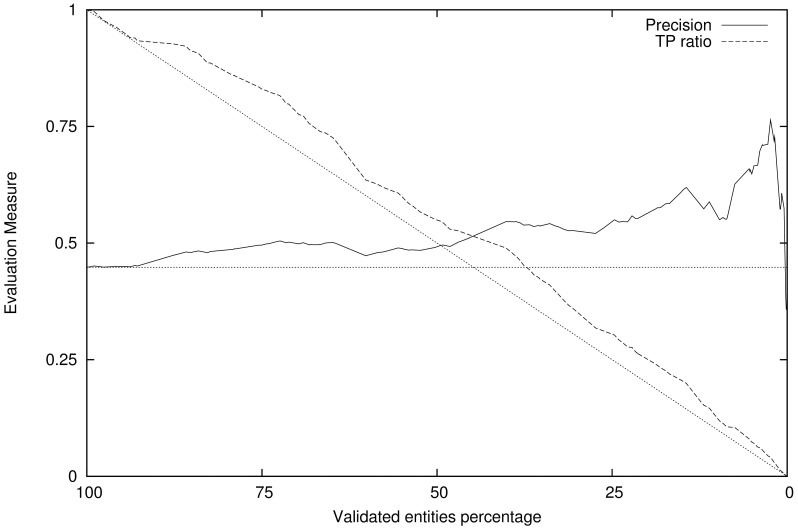
Validation of CRF-based annotation results. Shows the variation in precision and recall with the validation score threshold, using the Resnik measure with a document as text window. Straight dots represent the expected behavior of a random validation system.

The subset of consistent annotations can now be evaluated against the gold standard annotations, and new values for precision and recall can be calculated for this subset that misses the outlier annotations. In Figures 1 and 2 we show the effect of the variation of the validation threshold (i.e. the size of the validated entity subset, that ranges from all entities validated when the threshold is low to none when its large) and the precision evaluation measure for that validated entity subset, as well as the ratio of true positives still present in that subset. Figure 1 presents the results obtained using the dictionary-based entity identification method (Whatizit) and Figure 2 the results using the CRF-based method. For both Figures the semantic similarity measure being used is, as an example, Resnik’s measure.

If we were to randomly select a subset from the entities provided by an entity identification system, the amount of true positives in that random selection would decay linearly. Similarly, the precision of entity recognition for a random selection would be constant and equal to the full set of annotations. Unlike in a random subset selection, using our validation score significantly increases the precision as we select a subset of entities with higher validation score. Also, the true positive ratio for a selection using our validation score is higher than for a random selection, which means our method is being able to discern between true chemical entities and entities that have mistakenly been annotated as chemical, preferentially maintaining the true positives and discarding the false ones.

Table 2 provides the results using different validation score thresholds, corresponding to subsets of validated entities consisting of 25%, 50% and 75% of the total automatic annotations, for each one of the three tested semantic similarity measures. We can see that the precision for the subsets using our method is higher than the precision of the entire set of annotations before our method was applied (results in Table 1). Analyzing the results presented in Table 2 we conclude that several semantic similarity measures may be successfully used. Both the Resnik and simGIC measures are dependent upon Information Content (IC) calculations while simUI is a more straightforward measure, however the three tested measures provided similar results.

**Table 2:** Validation results, using the document as text window.

Subset		25% entities validated		50% entities validated			75% entities validated	
Measure	Method	TP	Precision		TP	Precision	TP	Precision
2*SimGIC	Dict	1,584	34.0		3,117	33.7	4,186	30.0
	CRF	1,361	51.1		2,781	52.1	3,761	46.9
2*SimUI	Dict	1,424	30.0		2,782	29.5	4,017	28.1
	CRF	1,335	49.8		2,632	49.3	3,781	47.6
2*Resnik	Dict	1,443	30.4		3,334	35.5	4,371	31.2
	CRF	1,449	55.0		2,633	49.0	3,968	49.6

Amount of True Positives (TP) and Precision obtained at selected subsets of validated entities corresponding to 25%, 50% and 75% of the total amount on annotations performed by each tool (Method), using validation calculated using the semantic similarity measure indicated in Measure. For this evaluation was used the document-wide as text window.

The dictionary method ranges from a precision of 24% for the total of annotations to about 30% precision when a quarter of the annotations that have lower validation score are discarded (a subset selection of 75% of the automatic annotations). This means an absolute increase of 6% precision, which corresponds to an increase of 25% relative to the original precision, without our method. The cost for this precision increase was the loss of 5% of the true positive annotations identified. Note that a random selection of 75% of the automatic annotation would maintain the precision at the same values (no relative increase) while the amount of true positives would decay by 25%.

For the CRF-based method, and when using a validated subset of the same size (75% of the automatic annotations), we see that the gain in precision is in the order of 5%, which corresponds to a relative precision increase of 11%. The cost in terms of true positive loss is in this case about 17%. The validation results in terms of ratio of true positives and increase of precision relative to the baseline results presented in Table 1 are provided in Table 3. We can clearly observe that the dictionary method benefits more from the validation method than the CRF-based method. This is most probably due to the starting precision of the two methods, which is higher for the CRF-based method, making it harder to discriminate correct annotations from annotation errors.

**Table 3:** Relative validation results, using the document as text window.

Subset		25% entities validated		50% entities validated		75% entities validated	
Measure	Method	TP Ratio	P Increase	TP Ratio	P Increase	TP Ratio	P Increase
2*SimGIC	Dict	35.0	39.9	68.8	38.7	92.4	23.5
	CRF	28.5	14.1	58.1	16.3	78.6	4.7
2*SimUI	Dict	31.4	23.5	61.4	21.4	88.7	15.6
	CRF	27.9	11.2	55.0	10.0	79.1	6.3
2*Resnik	Dict	31.8	25.1	73.6	46.1	96.5	28.4
	CRF	30.3	22.8	55.0	9.4	83.0	10.7

For selected subsets of validated entities corresponding to 25%, 50% and 75% of the total amount on annotations performed by each tool (Method), we present the True Positive ratio (TP Ratio), which corresponds to the percentage of True Positives remaining in the subset, and the Precision increase relative to the Precision for the total amount on annotations (P Increase). For this evaluation was used the document-wide as text window.

**Figure 3:**
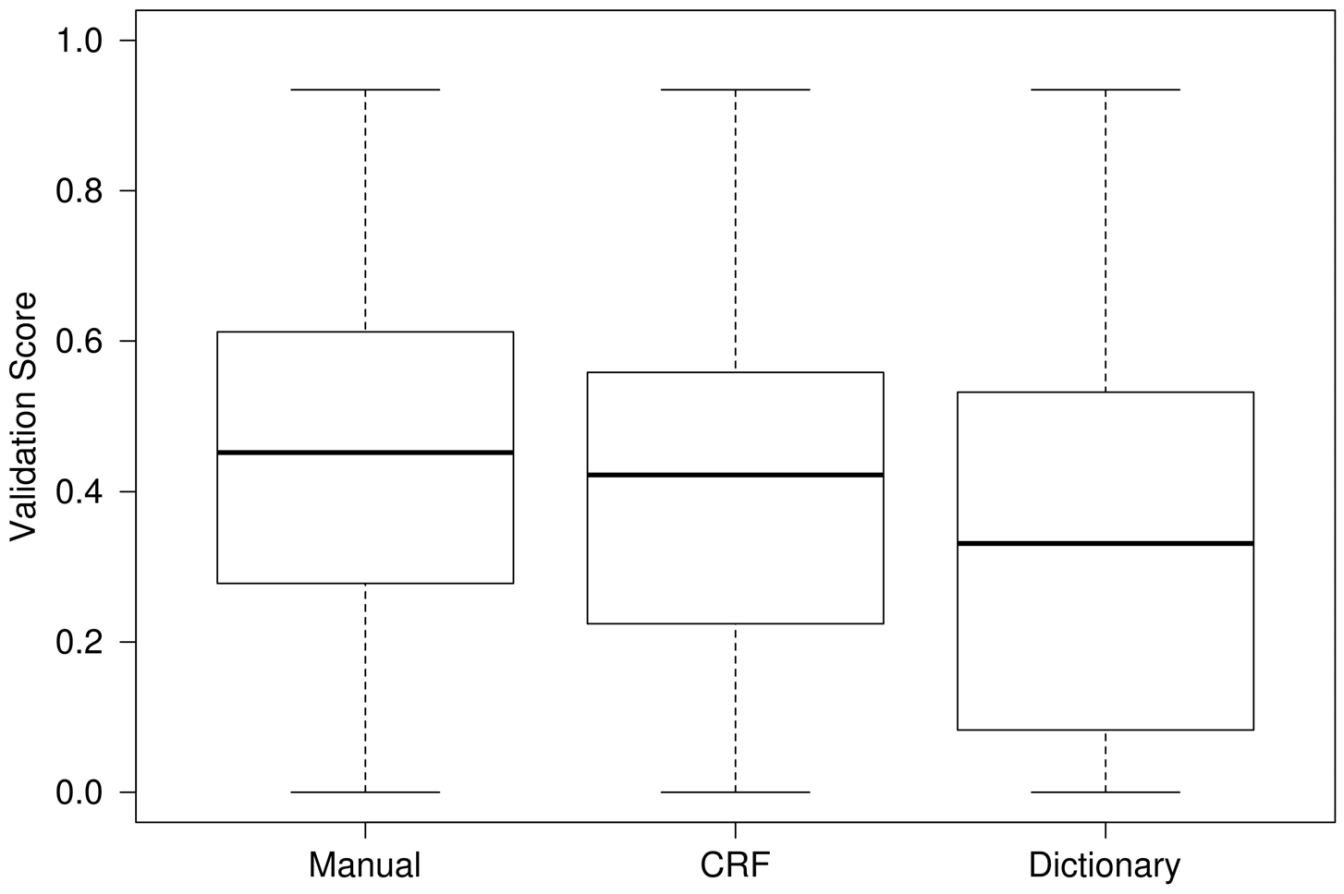
Comparison of the validation scores. Boxplot of the validation score obtained for the manual annotations in the gold standard, and the automatic annotations provided by the dictionary-based method and the CRF-based method.

In addition to the automatic annotations provided by the two entity recognition and resolution systems, we have also the manual annotations in the patent document gold standard, which are considered the ground truth. We applied our method to those annotations and compared their validation score distribution with that of the automatic annotation obtained by the two entity recognition systems. Figure 3 provides a boxplot with such comparison, where it can be observed that the manual annotations obtained higher validation scores than the automatic annotations. Between the automatic annotations, the dictionary-based method obtained lower validation score values than the CRF-based method. This indicates that the quality of the starting annotations has an impact in the obtained validation scores, which are higher for better quality starting annotations. The validation method proved efficient with different degrees of quality of the starting annotation, and good starting results can still profit from our method.

The validation results presented until now have considered the entire document as text window for validation score calculation, and each instance of a compound had a single validation score that was the similarity of the most similar compound in the document. However there might be large documents that change its scope in different sections, and thus the same compound should have different validation scores according to its position. This is why the calculation of the validation scores can be made using not only document-wide text windows, but also smaller ones such as paragraph-wide or even sentence-wide text windows. In this case a document will not be represented by single set of compounds, but a set of compounds for each text window and the validation scores are calculated comparing the entities in each of these windows.

In Table 4 we show the results using a paragraph-wide validation. We see that in this case there is usually a loss in performance when comparing with the document-wide validation, with the exception of a an increase from 34% precision to 38% when using a subset of 25% of the entities and the simGIC similarity measure for the dictionary-based annotations.

**Table 4:** Validation results, using the paragraph as text window.

Subset		25% entities validated			50% entities validated			75% entities validated	
Measure	Method		TP	Precision		TP	Precision	TP	Precision
2*SimGIC	Dict		1,650	36.6		2,871	31.6	3,879	28.3
	CRF		1,349	53.6		2,617	51.2	3,722	48.8
2*SimUI	Dict		1,644	36.0		2,820	30.6	3,858	28.2
	CRF		1,363	55.3		2,593	51.1	3,531	46.7
2*Resnik	Dict		1,606	35.3		3,075	34.5	4,047	29.6
	CRF		1,520	60.1		2,696	52.9	3,799	49.5

Amount of True Positives (TP) and Precision obtained at selected subsets of validated entities corresponding to 25%, 50% and 75% of the total amount on annotations performed by each tool (Method), using validation calculated using the semantic similarity measure indicated in Measure. The text window used for this evaluation was a paragraph-wide text window.

**Figure 4:**
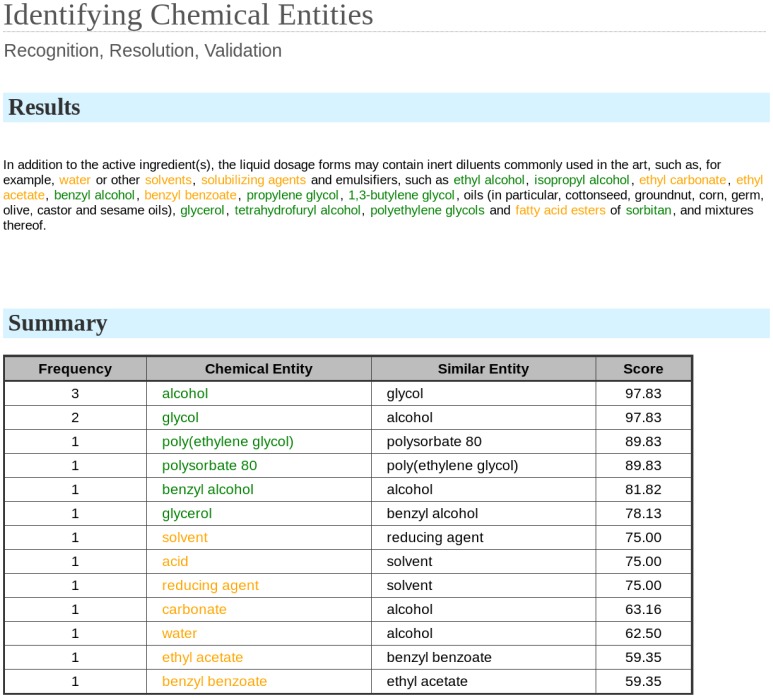
Web tool screenshot. Our validation method was implemented in a web tool containing the CRF-based chemical identification method. A sample sentence from patent document WO2007041564 is shown with some chemical entities, and their validation score. Validated entities (validation score above the defined validation threshold, selected in this example as 75%) are presented in green, while outliers are presented in yellow.

The method here presented has been implemented in a freely available web tool (www.lasige.di.fc.ul.pt/webtools/ice/) which integrates the CRF-based entity recognition method and the lexical similarity entity resolution method together with the presented validation method. A screenshot of the tool is presented in Figure 4.

### Examples

We analyzed the annotations with high validation scores, which are expected to be true positives, and found that most of them were in fact true positives. The entities with highest validation score are usually very related pairs and far from the root of the ontology. Examples are for instance “sodium hydroxide” and “potassium hydroxide”, “trichloroethanol” and “2-chloroethanol” or “chloroform” and “dichloromethane” that have been correctly validated with a high validation score. Some related entities such as “cetoleic acid” and “erucic acid” that not only have similar structures but also similar roles have obtained very high validation scores, while other structurally very similar entities such as “D-amino acids” and “L-amino acids” had lower validation scores.

#### Missing in ChEBI

Although most of the high validation score annotations were true positives, some did not match the gold standard manual annotations. For instance, we found that for both automatic entity identifications systems the terms “cyfluthrin”, “transfluthrin”, “flucythrinate”, “bioallethrin” and some others, all located in the same sentence of the patent document WO2007005470, contained a high validation score and were true positives. Analyzing that sentence we find that it is listing a series of pyrethroid insecticides, and its also because of that matching biological role that the validation score is very high for those entities.

However, an opposite example can also be found in that same sentence. The terms “bifenthrin”, “cyperaiethrin”, “methothrin” and “metofluthrin” have also been annotated as being chemical terms by the CRF-based method but failed to be mapped to ChEBI. Investigating those compounds we found that they were also pyrethroid insecticides, but had not yet been included in ChEBI. This is an example of an interesting aid our method can provide to curators or other users of chemical name recognizers that provide identification of putative chemical entities, not included yet in databases.

#### Missing in gold standard

Most of the entities identified as chemicals with a very high validation score and that were not tagged as chemicals in the gold standard (and thus were considered false positives) were found to be in fact chemical entities that for some reason had escaped manual annotation of the gold standard corpus. Examples of terms in this situation include “amino acid”, “peptide” and “aryl”. It is also interesting to note that usually the terms in this situation have been found multiple times in the same patent document. For instance, “amino acid” in the patent document WO2007041240, “peptide” in the patent document WO2007002913, and “aryl” in the document WO2007004952.

Among the annotation errors detected within high similarity score terms, we also found as an example the following sentence in the patent document WO2007045478 of the corpus:

“Compounds of the invention may further be useful for the treatment of withdrawal symptoms caused by termination of the use of addictive substances, like heroin, cocaine, tobacco, nicotine, opioids, benzodiazepines and alcohol.”

In this sentence, the dictionary method annotates heroin, cocaine, nicotine and alcohol. The CRF-based method annotates benzodiazepines in addition to those terms found by Whatizit. The similarity score of the terms is high, because the terms are closely related in ChEBI, but they are considered annotation errors. The reason is that the manual annotation of the corpus did not consider those terms. This is another example that shows that the gold standard might have been under annotated by the curators, and many false positive automatic annotations might in fact be correct annotations that have not been considered in the process of manual annotation of the corpus.

#### Adverse context

On the other end, for low validation score terms we find examples of situations where low score terms are in fact correctly identified. For instance, in the following sentence of the patent document WO2007041479:

“A pharmaceutical composition comprising (i) talnetant, (ii) povidone, (iii) mannitol and (iv) a surfactant, wherein: (a) the ratio of povidone to mannitol is 0.45∶1 or higher.”

Several terms were correctly identified as chemical, and the resolution to ChEBI has been correctly performed, but the terms simply had a low semantic similarity between them. This occurs because in this sentence the author was listing a pharmaceutical composition, and the compounds did not need to have any relationship between them other than being part of that composition. This type of error will tend to be less frequent as the size of the comparison window increases.

Also, the presence of words such as “comprise”, “compose”, “constitute” or other synonyms, might be used to assume a relation between the mentioned chemical entities even if their base validation score is low, so that validation score can be tweaked to allow for a correct validation of the entities.

### Conclusions

In this paper we proposed a method for validation of automatic chemical entity identification results that improves the precision of chemical entity identification tasks for state-of-the-art tools. This is because our method uses the fact that chemical entities named nearby, in a text window, have an intrinsic relationship that may be found on an ontology such as ChEBI. Text mining tools do not consider this by themselves, and thus our method can aid in their task of efficiently identifying chemical entities.

To demonstrate the feasibility of our method we have used the results of two distinct chemical entity identification methods, using a corpus that had also been manually annotated. Applying our method to the results of those two chemical identification methods, we were able to efficiently discern between true and false positive entities, enriching the precision obtained for entity identification in subsets of consistent entities. This is done by using semantic similarity measures in the ChEBI ontology to compare the chemical entities found in the text, and assign them a validation score. The high scoring entities are considered consistent while the low scoring entities are considered outliers and not validated.

The size of the set of validated entities is tunable by a validation threshold, and also by the type of semantic similarity measure and aggregate function implemented. This allows for fine tuning by manual curators that can use our method as a tool to give them assistance.

There are still some improvements that can be included in our method. For instance, we found that in some sentences that list components of a mixture, the individual compounds of that mixture do not need to be related, and thus the validation score is low for those compounds even though they are true positives. This issue can be dealt by detecting keywords such as “constitute” or “compose” that are an indication that the compounds in the vicinity do not need to be related, and tune their validation score with this fact in mind.

Our method can be applied to all chemical entity recognition tools that perform resolution to ChEBI, and has been implemented in a web tool on top of the CRF-based entity recognition method here used. Furthermore, our method can be easily adapted to other entity types than chemicals, given that there is an ontology available to compare those entity types and recognition tools that provide mappings to that ontology.

Beyond entity recognition we believe that our validation method can also be useful for relation extraction, since high semantic similarity in ChEBI for pairs of recognized entities may provide a strong evidence for predicting a relation between them. For example, in the sentence “paracetamol acts as a COX inhibitor”, the terms paracetamol (CHEBI:46195) and COX inhibitor (CHEBI:35544) are strongly connected in the in the *role* branch of ChEBI, thus our validation method would have provided a high score for this pair.

We used a system that uses CRF models based on a manually annotated patent document corpus to locate the chemical terms [19]and a lexical similarity method to perform resolution of those terms to ChEBI [20].

## Methods

We developed a validation method that receives as input the result of any chemical entity recognition tool that performs resolution to ChEBI. To test our method we used two systems that were previously presented: the dictionary-based method Whatizit [18] and a machine-learning approach that uses CRF models based on a manually annotated patent document corpus to locate the chemical terms [19] and a lexical similarity method to perform resolution of those terms to ChEBI [20]. However, any system that can provide chemical entity recognition and resolution to ChEBI may be used.

The output of our method is the list of the chemical entities ranked by their validation score that is calculated through its semantic similarity to the other chemical entities identified in a given text window. This score corresponds to a measure of similarity between the target entity and those found nearby. Thus, the basic idea behind the method is that related entities found together have a bigger chance of being true positive annotations than entities that do not have any significant relatedness with other entities found in the same text window.

### Entity Annotations

The input of our method is the text processed by any chemical entity identification tool that can provide resolution to ChEBI. The text can correspond to a full document, or be smaller text windows such as paragraphs or sentences. The text used represents the window where the similarity of one concept with the others in that window will be calculated.

Thus, the input for our method is the set of concepts of a reference database, e.g. ChEBI, that are mapped with the entities identified in a given text window 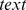:
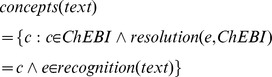



### ChEBI Semantic Similarity

Chemical Entities of Biological Interest (ChEBI) is a freely available dictionary and ontology of small molecular entities [2]. The ChEBI ontology structure comprises three separate sub-ontologies: the Chemical Entity sub-ontology provides a structural relationship between terms; the Role ontology provides a functional relationship between terms; the Subatomic Particle contains the entities which are smaller than the atom and its relations. ChEBI contains however several cyclic relationship types (such as “is enantiomer of”) that had to be removed from the ontology, remaining only non-cyclical relationships. The result is a directed acyclic graph (DAG) structure similar to that of GO and that supports the calculation of semantic similarities in a similar fashion.

Our method requires the ChEBI ontology for the calculation of the semantic similarity between two concepts:
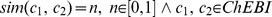



In our study, the January 2013 version of ChEBI was used, and we selected the Resnik, simUI and simGIC semantic similarity measures [26] given their previous successful application in ChEBI [27]. To avoid a bias to any given corpus, we used an intrinsic IC measure where the frequency of a term is proportional to the number of child terms it has in the ontology. Thus, the root term which is the most generic term will have the lowest IC while leaf terms far from the root are the most specific and have the highest IC. This IC calculation allows for independence upon a corpus and will adapt as the ontology grows.

### Validation Scores

For each concept our method calculates the similarity between it and all the other concepts in the given text window. Thus, an aggregate function is required to return a single score value from all its similarities:
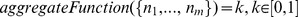



In this paper, the function used to aggregate the similarity measures of a concept with the other concepts in the text window is the maximum. Thus,  in this case corresponds to 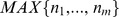 which represents the value of semantic similarity between a concept 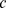 and the most similar concept to 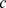 in the text window.

This is a straightforward approach that provides high values of 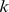 for concepts that have at least one similar concept in the text window, and low score for those that do not have at least one similar concept in the text window. However in some situations different ways to calculate the validation score might be beneficial and thus different aggregate functions can be used. As an example, other functions different from the maximum similarity measure may include the average of the similarity measures of one concept with all the others in the text window, or the average of the top 3 similarity measures for a concept in the text window.

Thus, a validation score can be calculated for each concept 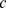 in the input text as follows:
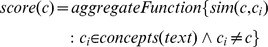



### Validation Threshold

With the validation score for each concept in a text window our method must decide which concepts (mapped entities) are to be validated and which are to be considered outliers.

The idea is that the top scoring entities are better candidates of being true positive annotations, while the lower scoring ones are better candidates to be false positives. Thus, our method ranks the entities according to their validation score.

The user can then provide a validation threshold 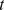 which is used to validate the entities for which the similarity score is higher than the given validation threshold. The remaining entities that contain a validation score lower than the validation threshold are considered outliers, because those entities do not present a significant semantic relationship to at least one other concept:
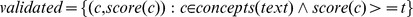


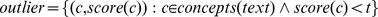



The validation threshold can also be automatically selected by deciding the number of entities to be validated. For example, if the user wants 25% of the entities to be validated the threshold 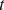 is given by the concept with the minimum score that is higher than 75% (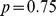) of all the other scores calculated: